# Kinetics of immune responses to SARS-CoV-2 proteins in individuals with varying severity of infection and following a single dose of the AZD1222

**DOI:** 10.1093/cei/uxac009

**Published:** 2022-01-27

**Authors:** Deshni Jayathilaka, Chandima Jeewandara, Laksiri Gomes, Tibutius Thanesh Pramanayagam Jayadas, Achala Kamaladasa, Gayasha Somathilake, Dinuka Guruge, Pradeep Darshana Pushpakumara, Thushali Ranasinghe, Inoka Sepali Aberathna, Saubhagya Danasekara, Buddini Gunathilaka, Heshan Kuruppu, Ananda Wijewickrama, Ruwan Wijayamuni, Lisa Schimanski, T K Tan, Graham S Ogg, Alain Townsend, Gathsaurie Neelika Malavige

**Affiliations:** 1 Allergy Immunology and Cell Biology Unit, Department of Immunology and Molecular Medicine, University of Sri Jayewardenepura, Nugegoda, Sri Lanka; 2 Colombo Municipal Council, Colombo, Sri Lanka; 3 National Institute of Infectious Disease, Sri Lanka; 4 MRC Human Immunology Unit, MRC Weatherall Institute of Molecular Medicine, University of Oxford, Oxford, UK; 5 Centre for Translational Immunology, Chinese Academy of Medical Sciences Oxford Institute, University of Oxford, Oxford, UK

**Keywords:** immune responses, SARS-CoV-2 proteins, natural infection, AZD1222

## Abstract

To characterize the IgG and IgA responses to different SARS-CoV-2 proteins, we investigated the antibody responses to SARS-CoV-2 following natural infection and following a single dose of AZD1222 (Covishield), in Sri Lankan individuals. The IgG and IgA responses were assessed to S1, S2, RBD, and N proteins in patients at 4 weeks and 12 weeks since the onset of illness or following vaccination. Antibodies to the receptor-binding domain of SARS-CoV-2 wild type (WT), α, β, and λ and ACE2 (Angiotensin Converting Enzyme 2) receptor blocking antibodies were also assessed in these cohorts. For those with mild illness and in vaccines, the IgG responses to S1, S2, RBD, and N protein increased from 4 weeks to 12 weeks, while it remained unchanged in those with moderate/severe illness. In the vaccines, IgG antibodies to the S2 subunit had the highest significant rise (*P* < 0.0001). Vaccines had several-fold lower IgA antibodies to all the SARS-CoV-2 proteins tested than those with natural infection. At 12 weeks, the haemagglutination test (HAT) titres were significantly lower to the α in vaccines and significantly lower in those with mild illness and in vaccines to β and for λ. No such difference was seen in those with moderate/severe illness. Vaccines had significantly less IgA to SARS-CoV-2, but comparable IgG responses those with natural infection. However, following a single dose vaccines had reduced antibody levels to the VOCs, which further declined with time, suggesting the need to reduce the gap between the two doses, in countries experiencing outbreaks due to VOCs.

## Teaser

Those with mild infection, vaccinees (AZD1222) and unexposed individuals had similar antibody levels to S2. Those with mild infection had highest antibody responses to S2, while hose with moderate/severe illness had highest responses to the receptor binding domain. Vaccinees had several fold lower IgA antibodies to all the SARS-CoV-2 proteins tested than those with natural infection.

## Introduction

The COVID-19 pandemic due to the SARS-CoV-2 virus continues to cause significant mortality and morbidity and many countries are experiencing a worse situation than experienced at the beginning of the pandemic [1]. The emergence of SARS-CoV-2 variants of concern such as the B.1.1.7 (α) and more recently B.1.617.2 (λ) has led to the exponential increase in the number of COVID-19 cases and deaths in many countries [1–3]. While the higher income countries have vaccinated a large proportion of their population, resulting in lower case numbers, many lower income and lower-middle income countries are grappling with the increase in the case loads, overburdening of health care resources and the inability to secure adequate doses of COVID-19 vaccines [4].

Although the duration of protection against re-infection from SARS-CoV-2 is not known, it has been shown that re-infection does occur, especially among older individuals, probably due to waning of immunity [5]. Re-infection has shown to occur particularly with certain variants such as P.1 (γ) variant in Brazil despite a very high seroprevalence [6], and also with B.1.351 (β) due to escape natural and vaccine-induced immunity [7]. Individuals who had experienced milder illness have shown to have reduced levels of neutralizing antibodies compared to those who had severe illness [8,9]. Apart from the presence of neutralizing antibodies to the receptor-binding domain (RBD), antibodies specific to S2 and N protein of SARS-CoV-2 are also detected in patients who have recovered from COVID-19 [10]. However, the usefulness of antibodies directed against S1, S2, and N protein in preventing re-infection is not known. The IgG and IgA specific to S1, S2 have been detected in the breast milk of infected mothers and, therefore, possibly provide protection to the neonate [11]. Antibodies against the S2 subunit have been detected in unexposed individuals and S1, S2, and N protein-specific memory B-cell responses have been detected in those who were infected with SARS-CoV-2 [12]. Children and adolescents who were unexposed to SARS-CoV-2 were shown to have a higher frequency of pre-exiting IgG antibodies specific to S2, which were able to cross neutralize SARS-CoV-2 [13]. The presence of high levels of cross-reactive antibodies to the S2 in children and adolescents has been speculated to reduce disease severity when infected with SARS-CoV-2 [13, 14]. Although many studies have investigated the role of SARS-CoV-2 specific IgG responses, virus-specific IgA was detected during early illness and was shown to be able to neutralize the SARS-CoV-2 virus to a greater extent than virus-specific IgG [15]. However, adults with severe illness had higher levels of SARS-CoV-2 specific IgA levels compared to adults with milder illness and children, which was shown to enhance disease severity *in vitro* by enhanced neutrophil activation and thus release of inflammatory mediators [16]. Therefore, although virus-specific IgA is an important component of mucosal immunity, its role in protection vs. disease pathogenesis is not clear. Further, the role of serum IgA, in contrast to mucosal IgA has not been studied extensively.

Currently, there are several vaccines for COVID-19, which have shown to be safe and have high efficacy rates against the original Wuhan SARS-CoV-2 virus and variants of concern [17–19]. However, due to the non-availability of an adequate quantity of vaccines and also to vaccinate as many individuals as fast as possible, some countries have increased the gap between the two doses of vaccine such as AZD1222 to 12 or 16 weeks [20]. While there have been many studies characterizing the IgG and IgA responses to different SARS-CoV-2 proteins in individuals with natural infection, the induction of IgG and IgA to different viral proteins in vaccines has not been extensively studied. It was recently shown that the mRNA vaccines induce high levels of both IgG and IgA antibodies against the spike protein [21]. However, there is limited data characterizing the IgG, IgA, ACE2-receptor-blocking antibodies in individuals with varying severity of the natural infection over time, in comparison to those who have received a single-dose of the AZD1222 vaccine. Therefore, in this study, we investigated the antibody responses in those with varying severity of natural infection and in those who received a single-dose of the AZD1222 at 4 weeks and 12 weeks to the S1, S2, RBD, and N proteins and also for SARS-CoV-2 variants of concern in a Sri Lankan population.

## Methods

### Patients

Patients confirmed SARS-CoV2 infection based on the positive RT-PCR who were admitted to the National Institute of Infectious Diseases (NIID), Sri Lanka, were recruited following informed written consent. They were followed throughout their illness while they were in hospital and clinical disease severity was classified as mild, moderate, and severe according to the WHO guidance of COVID-19 disease severity [22]. For this study, we recruited two cohorts of patients (Supplementary Table S1). Serum samples from the patient cohort 1 (*n* = 30) were used to determine the IgG and IgA antibody levels at 4 weeks since onset of illness, the ACE2 receptor-blocking antibody levels, and the antibodies to RBD by the HAT assay for the wild type (WT) and SARS-CoV-2 variants. The duration of illness was defined from the day or onset of symptoms and not the day of PCR positivity or admission to the hospital. Based on the WHO COVID-19 disease classification, 15 patients had mild illness and 15 patients had moderate/severe illness [22]. As all the patients in the first cohort could not be traced at 12 weeks, to carry out the above assays, we recruited a second cohort of patients. Based on the WHO COVID-19 disease classification, 14 patients had mild illness and 6 patients had moderate/severe illness [22].

To compare the antibody responses following infection with one dose of the AZD1222 vaccine, we recruited 20 individuals 4 weeks following vaccination and the same 20 individuals were followed at 12 weeks following vaccination. All 20 individuals who were included at 4 weeks following vaccination were included at 12 weeks following vaccination as well. We also included serum samples from individuals who had a febrile illness in 2017 and early 2018. Ethical approval was received by the Ethics Review Committee of Faculty of Medical Sciences, University of Sri Jayewardenepura. Informed written consent was obtained from patients.

### Luminex assay to measure SARS-CoV-2 S1, S2, RBD, and N specific IgA and IgG antibody responses

SARS-CoV-2 S1, S2, RBD, and N specific IgA and IgG antibody responses were measured separately using multiplex SARS-CoV-2 antigen panels IgG and IgA (Millipore). The assay was carried out according to manufactures instructions. The mean fluorescence intensity (MFI) was measured in each serum sample using MAGPIX® which was positively correlated with S1, S2, RBD, and N specific IgG and IgA in serum.

### Haemagglutination test (HAT) to detect antibodies to the receptor-binding domain (RBD)

The HAT was carried out as previously described [23]. The B.1.1.7 (N501Y), B.1.351 (N501Y, E484K, K417N), and B.1.617.2 versions of the IH4-RBD reagent were produced as described [23], but included the relevant amino acid changes introduced by site-directed mutagenesis. These variants were titrated in a control HAT with the monoclonal antibody EY-6A (to a conserved class 4 epitope [23, 24]) and found to titrate identically with the original version so 100ng (50 μl of 2 μg/ml stock solution) was used for developing the HAT. The assays were carried out and interpreted as previously described [25]. The HAT titration was performed using 11 doubling dilutions of serum from 1:20 to 1:20,480, to determine the presence of RBD-specific antibodies. The RBD-specific antibody titre for the serum sample was defined by the last well in which the complete absence of “teardrop” formation was observed.

### Surrogate neutralizing antibody test (sVNT) to detect NAbs

The surrogate virus neutralization test (sVNT) [26], which measures the percentage of inhibition of binding of the RBD of the S protein to recombinant ACE2 [26] (Genscript Biotech, USA) was carried out according to the manufacturer’s instructions as previously described by us [9]. Inhibition percentage ≥25% in a sample was considered as positive for NAbs.

### Statistical analysis

Data were analysed by GraphPad Prism 9 version 9.2.0. The data were first tested for normality and homoscedasticity using Shapiro Wilk and Levene’s tests and since the assumptions were violated, non-parametric tests were used for the analysis. Kruskal–Wallis test was used to determine the difference between the antibody levels between the three different groups (two-tailed) followed by multiple comparisons using the two-stage step-up procedure of Benjamini, Krieger, and Yekutieli while controlling the false discovery rate (FDR) Mann–Whitney test (two-tailed) was used to determine the differences between antibody levels between 4 weeks and 12 weeks in those with natural infection. Wilcoxon paired *t*-tests (two-tailed) were used to determine the differences between antibody titres against S1, S2, RBD, N proteins, and ACE2 receptors in vaccinated individuals and the antibody titres to WT, B.1.1.7 (α), B.1.351(β), and B.1.617.2 (λ) between 4 weeks and 12 weeks in both naturally infected and vaccinated individuals. The antibody titres were compared between the WT, B.1.1.7 (α), B.1.351 (β), and B.1.617.2 (λ) at both time points for both naturally infected and vaccinated using the Friedman test followed by multiple comparisons using the two-stage step-up procedure of Benjamini, Krieger, and Yekutieli while controlling the false discovery rate (FDR).

## Results

### The kinetics of SARS-CoV-2 specific IgG responses in those with natural infection

**Figure 1.**
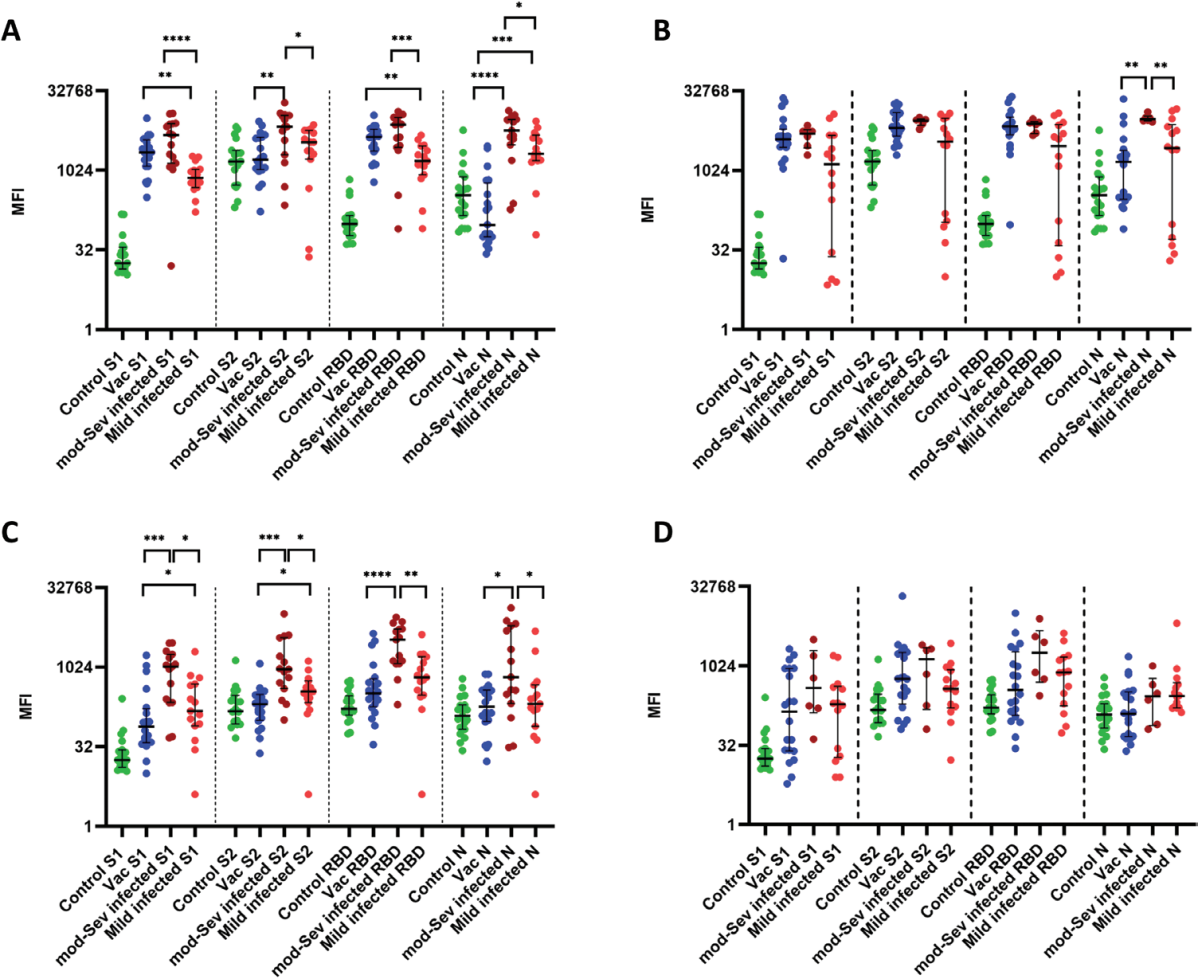
IgG and IgA antibody levels to S1, S2, RBD, and N protein of SARS-CoV-2 in individuals following natural infection and following a single dose of the AZD1222 vaccine. IgG antibodies to S1, S2, RBD, and N protein was measured by Luminex assays at 4 weeks in those with mild illness (*n* = 15), moderate/severe illness (*n* = 15), vaccines (*n* = 20) and controls (*n* = 19) (**A**) and again at 12 weeks in those with mild illness (*n* = 14), moderate/severe illness (*n* = 6), vaccines (*n* = 20) (**B**). IgA antibodies were also measured in the above groups at 4 weeks (**C**) and at 12 weeks (**D**). The Kurskal–Wallis test was used to determine the difference between the antibody levels between the three different groups (two-tailed) followed by multiple comparisons using two-stage step-up procedure of Benjamini, Krieger, and Yekutieli while controlling the false discovery rate (FDR). The lines indicate the median and the interquartile range.

IgG responses to the S1, S2, RBD, and N protein were measured in individuals with COVID-19 at 4 weeks and 12 weeks since the onset of illness and also in serum samples of 15 individuals who had a febrile illness in 2017 and early 2018. At 4 weeks since onset of illness, the highest magnitude of IgG antibody responses was seen for RBD in those with moderate/severe illness, whereas those with mild disease, had the highest responses to S2 (Fig. 1A, Table 1). Those who had a febrile illness in year 2017 and 2018 (controls), also had high antibody levels to S2, but not for other proteins. There was no difference in the antibody levels to S2 in those with mild illness compared to the controls (*P* = 0.213), although those with milder disease had significantly higher antibody levels to S1 (*P* = 0.002) and RBD (*P* = 0.0028) and N protein (*P* = 0.0044), than the controls. Further, those with moderate/severe infection had significantly higher antibody titres compared to mild illness to all the proteins (Fig. 1A). In those who received a single dose of the AZD1222 vaccine, the IgG responses to the S1 and S2 protein was similar, although the levels for the RBD was significantly higher (Table 1). As expected, the IgG responses to the N protein was very low, but even lower than for the controls. The antibody levels to S1 (*P* = 0.0002), S2 (*P* = 0.01), RBD (*P* = 0.002) and N (*P* < 0.0001) proteins were found to be significantly different between the three groups of individuals at 4 weeks as resulted by Kruskal–Wallis test (Fig. 1A).

**Table 1.** Antibody responses to S1, S2, RBD, and N protein of the SARS-CoV-2 in those with varying severity of illness and in those following a single dose of the AZD1222. MFI indicates the median fluorescence intensity.

	4 weeks Median (IQR)	12 weeks Median (IQR)	*P* value
Mild infection (IgG)			
S1	734 (483–1071)	1336 (24–4714)	0.59
S2	3503 (1656–5795)	3579 (106.8–9912)	0.68
RBD	539 (840-2960)	2952 (38.7–7516)	0.59
N	2094 (1554–4787)	2694 (51–7547)	0.84
Mild infection (IgA)			
S1	152 (79–490)	192 (19–422.1)	0.69
S2	354 (219–561.5)	380.2 (165.6–869)	0.71
RBD	656.5 (303–1616)	770.5 (180.3–1520)	0.98
N	207.5 (78–468)	276.3 (165.5––496.5)	0.31
Moderate/severe infection (IgG)			
S1	4776 (1395––7833)	5064 (2744–6038)	0.96
S2	6869 (2001––11 131)	8931 (7262–9607)	0.85
RBD	7486 (2784––10 218)	7829 (5083–8553)	0.67
N	5831 (3123––9383)	9538 (8810–10 844)	0.31
Moderate/severe infection (IgA)			
S1	1043 (220–1784)	391.8 (132.8–2021)	0.52
S2	934 (399–3679)	1378 (153.9–2269)	0.73
RBD	3375 (1192–5401)	1837 (506.1–4802)	0.38
N	661 (211.5–6165)	273 (75.9–596.1)	0.18
Vaccinated IgG			
S1	2215 (1223–3870)	3969 (2805–6199)	0.0003
S2	1625 (1063–4329)	6537 (4570–12 690)	<0.0001
RBD	4393 (2355–6131)	6983 (4817–10 421)	0.0002
N	95 (57–591)	1482 (290–2447)	<0.0001
Vaccinated IgA			
S1	76.5 (38.2–166.5)	140 (25–921)	0.363
S2	203.3 (101.3–310.9)	585 (194–1855)	0.0017
RBD	327.5 (183–612.8)	360 (119–1902)	0.956
N	182 (96–375)	127 (47–330)	0.622

At 12 weeks since the onset of illness, those with moderate/severe illness had the highest responses to N protein, whereas those with mild illness still had the highest responses to S2 (Fig. 1B). At 12 weeks for all proteins, those with moderate/severe disease had significantly higher antibody levels than those with milder illness (Fig. 1B). The antibody responses only to N protein (*P* = 0.0137) was significantly different between the those with mild illness, moderate/severe disease and the vaccines as resulted by Kruskal–Wallis test (Fig. 1B). From 4 to 12 weeks, the S1 and RBD specific antibodies rose in those with mild illness, although they were not significant (Table 1). Patients who had moderate/severe illness sustained the same levels of antibodies for all four proteins from 4 weeks to 12 weeks. In the vaccines, from 4 weeks to 12 weeks the IgG levels to S1 (*P* = 0.0003), S2 (*P* < 0.0001), RBD (*P* = 0.0002) and N (*P* < 0.0001) had significantly increased (Table 1).

### The kinetics of SARS-CoV-2 specific IgA responses in those with natural infection

IgA responses to the S1, S2, RBD, and N protein were measured in the above individuals with COVID-19 at 4 weeks and at 12 weeks since the onset of illness or following vaccination and also in serum samples of 15 individuals who had a febrile illness in 2017 and early 2018. At 4 weeks and 12 weeks of illness individuals with both mild and moderate/severe illness, had the highest levels of IgA antibodies to the RBD (Fig. 1C and D). However, those with moderate/severe disease had significantly higher antibody responses to all four proteins when compared to those with mild illness at 4 weeks, but there was no difference at 12 weeks (Table 1). Vaccines had similar responses to all four proteins, including the N protein at 4 weeks (Table 1). IgA levels for S1 (*P* = 0.004) and RBD (*P* = 0.0262) were significantly higher than the control group in the vaccines. However, at 4 weeks vaccines had significantly lower IgA levels to all proteins compared to those who had moderate/severe infection (Fig. 1C). Significant differences of IgA responses were seen in those with mild illness, moderate/severe illness, and vaccines for S1 (*P* = 0.001), S2 (*P* = 0.0003), RBD (*P* = 0.0003), and N protein (*P* = 0.04) at 4 weeks as resulted by Kruskal–Wallis test (Fig. 1C).

There was no difference in IgA levels to any of the proteins at 4 weeks compared to 12 weeks in patients with mild illness or with moderate/severe illness (Table 1). However, at 12 weeks, no significant differences were seen between the three groups to S1, S2, RBD, and N protein (Fig. 1D).

### ACE2 receptor blocking antibodies following natural infection and one dose of AZD1222

**Figure 2.**
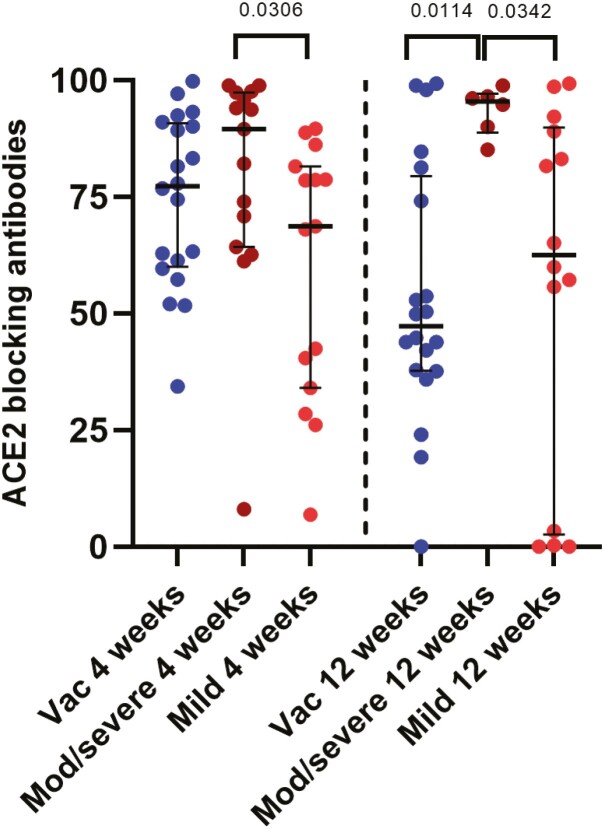
ACE2 receptor blocking antibodies in patients with varying severity of illness and following a single dose of the AZD1222 vaccine. ACE receptor blocking antibodies were measured by the surrogate virus neutralizing test following natural infection at 4 weeks in those with mild illness (*n* = 15) and moderate/severe illness (*n* = 15) and at 12 weeks in those with mild (*n* = 14) and moderate/severe illness (*n* = 6). Antibodies were also measured at 4 weeks (*n* = 20) and 12 weeks (*n* = 20) in vaccines following a single dose of AZD1222. The Kurskal–Wallis test was used to determine the difference between the antibody levels between the three different groups (two-tailed) followed by multiple comparisons using two-stage step-up procedure of Benjamini, Krieger, and Yekutieli while controlling the false discovery rate (FDR). The lines indicate the median and the interquartile range.

Due to the lack of BSL-3 facilities to measure neutralizing antibodies, we used a surrogate test to measure the inhibition of binding of antibodies in patient sera to the ACE2 receptor [26]. This was shown to be 100% specific in the Sri Lankan population, with none of the sera of individuals collected in 2017 and 2018, giving a positive response [9]. The ACE2 blocking antibodies were significantly higher in those with moderate to severe illness when compared to those with mild illness at 4 weeks (*P* = 0.0306) and at 12 weeks (*P* = 0.0342) as reported previously (Fig. 2) [9]. However, in those who received a single dose of the vaccine, the ACE2 blocking antibodies significantly reduced (*P* < 0.0001) from levels at 4 weeks (median 77.32, IQR 60.05–90.77% of inhibition) to 12 weeks (median 38.17, IQR 28.95–57.28% of inhibition).

### Antibodies to the receptor binding domain of the spike protein, including variants, measured by the haemagglutination test (HAT)

HAT is a surrogate test to detect SARS-COV-2 NAbs, with high sensitivity and specificity, that correlate with neutralizing activity [27]. We previously evaluated the usefulness of the HAT assay in determining antibody responses to the RBD of the SARS-CoV-2, wild type (WT) virus, B.1.1.7 (α) variant, and the B.1.351 (β) variants at 4 weeks following a single dose of the AZD1222 vaccine and had also evaluated this assay in naturally infected individuals in Sri Lanka [28]. In this study, we proceeded to investigate the differences in the antibody responses to the RBD in those with natural infection at 4- and 12-weeks following infection, and after a single dose of the AZD1222 vaccine. The antibody responses to the WT, B.1.1.7 (α), B.1.351 (β), and B.1.617.2 (λ) were measured.

**Figure 3.**
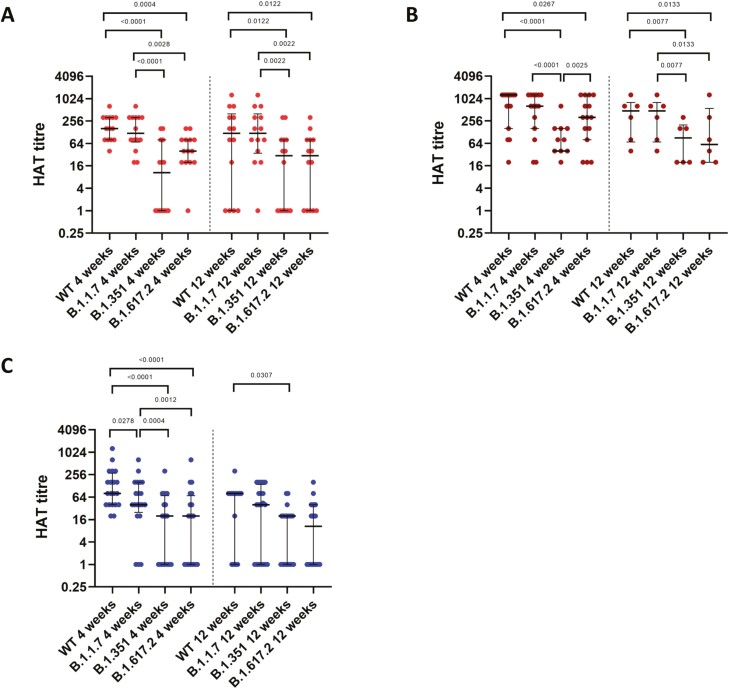
Comparison of antibody titres to RBD of the SARS-CoV-2 using the HAT assay in those with varying severity of infection and in vaccines. Antibody titres were measured in individuals with mild illness to the WT, B.1.1.7 (α), B.1.351 (β), and B.1.617.2 (λ) at 4 weeks (*n* = 15) and 12 weeks (*n* = 14) since the onset of illness (**A**), in those with moderate/severe illness at 4 weeks (*n* = 15) and 12 weeks (*n* = 6) since onset of illness (**B**) and in those who received one dose of AZD1222 vaccine at 4 weeks (*n* = 20) and 12 weeks (*n* = 20) following the vaccine (**C**). The difference between antibody titres to WT, B.1.1.7 (α), B.1.351 (β), and B.1.617.2 (λ) was determined using the Wilcoxon paired *t*-test (two-tailed). The lines indicate the median and the interquartile range.

In those with mild illness, at 4 weeks from the onset of the illness the median antibody titres to the WT was 160 (IQR 80–320), B.1.1.7 (α) was 120 (IQR 70–320), B.1.351 (β) was 10 (IQR, 0–80) and for B.1.617.2 (λ) it was 40 (IQR 20–80). The antibody titres for the WT was significantly higher compared to B.1.351 (β) (*P* < 0.0001) and B.1.617.2 (λ) (*P* = 0.0004) (Fig. 3A). At 12 weeks following the onset of illness, although there was a slight reduction in the antibody titres to the WT (*P* = 0.44) and B.1.617.2 (λ) (0.39), this was not statistically significant (Table 2). In those with moderate/severe illness at 4 weeks from the onset of illness the median antibody titres to the WT was 1280 (IQR 160–1280), B.1.1.7 (α) was 640 (IQR 160–1280), B.1.351 (β) was 40 (IQR 0–160) and for B.1.617.2 (λ) it was 320 (IQR 80–1280) (Fig. 3B). There was no significant difference between the antibody titres for the WT compared to B.1.1.7 (α) (*P* = 0.28), but for B.1.315 (β) (*P* < 0.0001) and B.1.617.2 (λ) (*P* = 0.004). Although the antibody titres for the WT and all the variants except B.1.351 (β) reduced from 4 to 12 weeks in those with moderate/severe illness, this was not statistically significant (Table 2).

**Table 2.** Antibody responses to WT, B.1.1.7 (α), B.1.351 (β), and B.1.617.2 (λ) variants of the SARS-CoV-2 in those with varying severity of illness and in those following a single dose of the AZD1222 measured by haemagglutination test (HAT).

	4 weeks Median (IQR)	12 week Median (IQR)	*P* value
Mild infection			
WT	160 (80–320)	120 (0–400)	0.4392
B.1.1.7	120 (70–320)	120 (35–400)	0.9548
B.1.351	10 (0–80)	30 (0–80)	0.5651
B.1.617.2	40 (20–80)	30 (0–80)	0.3947
Moderate/severe infection			
WT	1280 (160–1280)	480 (70–800)	0.2151
B.1.1.7	640 (160–1280)	480 (70–800)	0.4492
B.1.351	40 (0–160)	90 (20–200)	0.4373
B.1.617.2	320 (80–1280)	60 (20–560)	0.2622
Vaccinated			
WT	80 (40–280)	80 (0–80)	0.0018
B.1.1.7	40 (25–160)	40 (0–140)	0.3687
B.1.351	20 (0–70)	20 (0–20)	0.2593
B.1.617.2	20 (0–70)	10 (0–40)	0.1406

At 4 weeks following a single dose of the vaccine, the median antibody titres to the WT was 80 (IQR 40–280), B.1.1.7 (α) was 40 (IQR 25–160), B.1.351 (β) was 20 (IQR 0–70) and for B.1.617.2 (λ) it was 20 (IQR 0–70) (Fig. 3C). At 12 weeks following a single dose of the vaccine, the antibody titres for WT was 80 (IQR 0–80), for B.1.1.7 (α) it was 40 (IQR 0–140), for B.1.351 (β) it was 20 (0–20) and for B.1.617.2 (λ) it was 10 (IQR 0–40) (Fig. 3C). From 4 to 12 weeks, although there was no significance difference of the antibody titres of the RBD of the B.1.1.7 (α) (*P* = 0.37), B.1.351 (β) (*P* = 0.26), and B.1.617.2 (λ) (*P* = 0.14), the antibody titres to WT significantly reduced (*P* = 0.0009) (Table 2). As previously described by us at 4 weeks following vaccination, the HAT titres were significantly lower for B.1.1.7 (α) (*P* = 0.0278), B.1.351 (β) (*P* < 0.0001), and for B.1.617.2 (λ) (*P* < 0.0001) compared to WT. However, there was no significance difference in antibody titres between B.1.351 (β) and B.1.617.2 (λ) (*P* = 0.0522) (Fig. 3C). At 12 weeks only B.1.351 (β) HAT titres were significantly lower than the WT (*P* < 0.0307).

**Figure 4.**
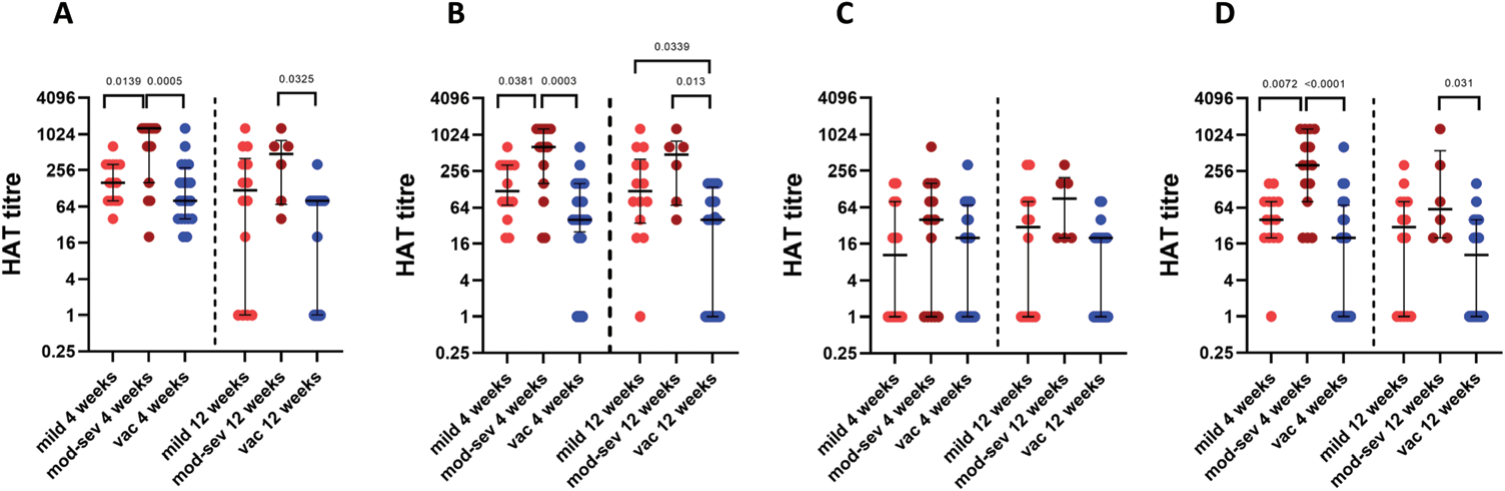
Comparison of antibody titres to the RBD of the SARS-CoV-2 using the HAT assay for the wild type and for variants. Antibody titres were measured in patients with mild illness (*n* = 15), moderate/severe illness (*n* = 15) from 4 weeks since onset of illness and in those who received one dose of AZD1222 vaccine at 4 weeks (*n* = 20), and again at 12 weeks in those who developed mild illness (*n* = 14), moderate/severe illness (*n* = 6) and in those who received 1 dose of AZD1222 vaccine (*n* = 20), for the WT (**A**), B.1.1.7 (α) (**B**), B.1.351 (β) (**C**), and B.1.617.2 (λ) (**D**). The Kurskal–Wallis test was used to determine the difference between the antibody levels between the three different groups (two-tailed) followed by multiple comparisons using two-stage step-up procedure of Benjamini, Krieger, and Yekutieli while controlling the false discovery rate (FDR). The lines indicate the median and the interquartile range.

Antibodies to the RBD were significantly different between those with mild illness, moderate/severe illness and with those with a single dose of the vaccine at 4 weeks (*P* = 0.004) and at 12 weeks (*P* = 0.02) for WT. At 4 weeks moderate/severe illness patients had significantly higher antibody titres to WT compared to those who had a mild illness (*P* = 0.0139) and those who were vaccinated (*P* = 0.0005) (Fig. 4A). This difference was also seen for the B.1.1.7 (α) at 4 weeks between those with mild illness and moderate/severe illness (*P* = 0.0381) and with those with moderate/severe illness and a single dose of the vaccine (*P* = 0.0003). However, at 12 weeks those with a single vaccine, had significantly low antibody titre compared to mild illness (*P* = 0.0339) and moderate/severe illness (*P* = 0.013) (Fig. 4B). Similarly, antibody titres against B.1.617.2 (λ) too were higher in patients with moderate/severe illness compared to mild illness (*P* = 0.0072) and vaccinated individuals with a single dose (*P* < 0.0001). At 12 weeks those with moderate/severe illness had higher antibody titre only against vaccinated individuals (*P* = 0.031) (Fig. 4D). However, there was no difference between the antibody titres to the B.1.351 (β) between those with mild, moderate/severe illness and vaccines at 4 weeks and 12 weeks (*P* = 0.02) (Fig. 4C).

## Discussion

In this study, we investigated the kinetics of IgG and IgA responses to S1, S2, RBD, and N protein, ACE2 receptor blocking antibodies and antibodies against SARS-CoV-2 variants, in individuals at 4 and 12 weeks following natural infection and in those who had a single dose of the AZD1222. Based on the Luminex assays for IgG and IgA levels to S1, S2, RBD, and N, IgG antibodies to these proteins following vaccination were increased significantly from 4 weeks to 12 weeks. In mild illness, although not significant, antibodies for S1 and RBD rose from 4 weeks to 12 weeks. In the vaccines, the most significant rise was seen for the S2 subunit, while in those with mild illness the rise was seen for IgG antibodies for the RBD. In those with moderate/severe illness while there was no change in the IgG responses from 4 to 12 weeks but the responses to the N protein had increased although this was not significant. Unexpectedly, the antibodies against N proteins were also increased from 4 to 12 weeks, possibly due to asymptomatic infection in some individuals after a single dose of the AZD1222 vaccine. Therefore, the kinetics of antibody responses to S1, S2, RBD, and N appear to vary based on the severity of the natural infection and also appeared to be different in vaccines. Interestingly, blood samples of those who had a febrile illness in 2017 and 2018 also gave IgG and IgA high responses to the S2 subunit, suggesting the presence of S2 subunit cross-reactive antibodies, in these donors as previously seen in other studies [13, 14]. Following a single dose of the AZD1222 vaccine, the antibodies against S2 appear to continue to rise from 4 to 12 weeks, possibly due to stimulation of pre-existing cross-reactive memory B cell responses to the S2 subunit [14].

SARS-CoV-2 specific IgA antibodies have been shown to be generated during early illness and have the potent neutralizing ability [15]. IgA antibodies to the RBD have been shown to develop earlier than IgG and while some studies have shown that serum IgA does not associate with clinical disease severity [15], patients who developed the severe disease were shown to have higher levels of virus-specific IgA [29]. Serum IgA was shown to activate neutrophils, thereby leading to the production of increased levels of inflammatory mediators leading to disease pathogenesis [16]. We found that at 4 weeks of illness, those with moderate/severe illness had significantly higher serum IgA to S1, S2, RBD, and N compared to those with mild illness, but these high levels of IgA declined except for S2 protein and there were no differences between these two groups at 12 weeks since the onset of illness. Vaccines had several fold lower IgA antibodies to all the SARS-CoV-2 proteins tested than those with mild and moderate/severe illness at 4 weeks and 12 weeks. The importance of serum IgA in preventing re-infection is currently unknown and if those with lower IgA have reduced protection is currently unknown.

Although the IgG antibodies to S1, S2, and the RBD rose from 4 to 12 weeks in the vaccines, the ACE2 receptor-blocking antibodies, which were shown to correlate with neutralizing antibodies significantly decreased [26]. The HAT assay, which also measures antibodies to the RBD and has shown to correlate well with the ACE2 receptor blocking assay and with neutralizing antibodies [23, 28], also showed that the RBD binding antibodies decreased from 4 to 12 weeks in the vaccines. This suggests that although ACE2 receptor blocking antibodies are reduced, the antibodies that bound to the S2 region increase which neutralizes the SARS-COV2 through inhibition of fusion and uncoating of the virus.

Apart from assessing antibodies to the RBD to the wild type, we assessed the antibodies to three other VOCs, B.1.1.7 (α), B.1.351 (β), and B.1.617.2 (λ). At 4 weeks following vaccination, the vaccines had a significantly lower levels of antibodies to the RBD of WT, B.1.1.7 (α), and B.1.617.2 (λ) compared to severe illness. The antibody levels among vaccines were significantly lower for B.1.1.7 (α), B.1.351 (β), and 1.617.2 (λ) compared to WT, showing a reduction in antibody binding to the RBD of the VOCs. These levels further declined at 12 weeks following vaccination, to VOCs, showing that a single dose of the AZD1222 was likely to offer less protection against VOCs. In fact, it has been shown that one dose of AZD1222 is only 33% effective in preventing symptomatic disease with B.1.617.2 (λ), 3 weeks following the first dose [30]. The efficacy of a single dose against B.1.617.2 (λ) is likely to decline further by 12 weeks, as the antibodies to RBD further wanned. However, the efficacy of two doses of AZD1222 against hospitalization was 92%, while for Pfizer-BioNTech was 96% [31]. Therefore, in countries that have outbreaks due to VOCs, especially B.1.617.2 (λ), it would be prudent to reduce the gap between the two doses to increase efficacy as currently carried out in many countries. Interestingly, although those with mild or moderate/severe illness also had a marked reduction in antibodies to the RBD of B.1.351 (β), they had higher levels of antibodies to the RBD of B.1.617.2 (λ) at 4 weeks compared to B.1.351 (β). However, by 12 weeks the antibody levels to both B.1.351 (β) and B.1.617.2 (λ) were similar. Therefore, B.1.617.2 (λ) had less immune evasion than B.1.351 (β) in those who were naturally infected, at least during early convalescence.

In summary, we have investigated the kinetics and differences in IgG and IgA antibody responses to the S1, S2, RBD, and N in those with varying severity of infection and vaccines who received a single dose of AZD1222, which showed that vaccines had significantly less IgA to SARS-CoV-2, but comparable IgG responses those with natural infection. However, following a single dose vaccines had reduced antibody levels to the VOCs, which further declined with time, suggesting the need to reduce the gap between the two doses, in countries experiencing outbreaks due to VOCs.

## Supplementary Material

uxac009_suppl_Supplementary_Table_S1

## Data Availability

All data are available within the manuscript, figures and the tables. Individual data points are shown in all figures. Source data are provided with this paper.

## Abbreviations

WT: wild type
HAT: haemagglutination test
VOC: variants of concern
RBD: receptor binding domain
sVNT: surrogate virus neutralization test
