# Supplementary material for: Kinetics of immune responses to SARS-CoV-2 proteins in individuals with varying severity of infection and following a single dose of the AZD1222
Source: Clin Exp Immunol. 2022 Jan 27;208(3):323–31. doi: 10.1093/cei/uxac009 (PMC8807318; doi:10.1093/cei/uxac009)
Supplement: uxac009_suppl_Supplementary_Table_S1 [file uxac009_suppl_supplementary_table_s1.docx]

|  | Gender | Age (Mean) |
| --- | --- | --- |
| Mild illness (at 4 weeks) | Males (n=9)  Females (n=6) | 32  50 |
| Severe/Moderate illness (at 4 weeks) | Males (n=10)  Females (n=5) | 45  46 |
| Vaccinated (at 4 weeks) | Males (n=11)  Females (n=9) | 42  41 |
| Mild illness (at 12 weeks) | Males (n=4)  Females (n=11) | 50  33 |
| Severe/Moderate illness (at 12 weeks) | Males (n=3)  Females (n=3) | 41  50 |
| Vaccinated (at 12 weeks) | Males (n=11)  Females (n=9) | 42  41 |

**Supplementary table 1: Demographic information of study participants**
